# Mentalizing During Social Interaction: The Development and Validation of the Interactive Mentalizing Questionnaire

**DOI:** 10.3389/fpsyg.2021.791835

**Published:** 2022-02-17

**Authors:** Haiyan Wu, Bowen J. Fung, Dean Mobbs

**Affiliations:** ^1^Centre for Cognitive and Brain Sciences and Department of Psychology, University of Macau, Taipa, Macau SAR, China; ^2^Division of the Humanities and Social Sciences, California Institute of Technology, Pasadena, CA, United States; ^3^Computation and Neural Systems Program, California Institute of Technology, Pasadena, CA, United States

**Keywords:** mentalization, meta-cognition, theory of mind, meta-mentalizing, scale development, mind reading, ultimatum game

## Abstract

Studies have shown that during social interaction a shared system underlies inferring one’s own mental state, and the mental states of others – processes often referred to as mentalization. However, no validated assessment has been developed to measure second order mentalization (one’s beliefs about how transparent one’s thoughts are to others), or whether this capacity plays a significant role in social interaction. The current work presents a interactive mentalization theory, which divides these directional and second order aspects of mentalization, and investigates whether these constructs are measurable, stable, and meaningful in social interactions. We developed a 20-item, self-report interactive mentalization questionnaire (IMQ) in order to assess the different sub-components of mentalization: self–self, self–other, and other–self mentalization (Study 1). We then tested this scale on a large, online sample, and report convergent and discriminant validity in the form of correlations with other measures (Study 2), as well as correlations with social deception behaviors in real online interaction with Mturk studies (Study 3 and Study 4). These results validate the IMQ, and support the idea that these three factors can predict mentalization in social interaction.

## Introduction

Humans have a rich capacity to infer the mental states and thoughts of others (i.e., self–other mentalization), possess the ability to look inward to self-monitor and assess thought processes (i.e., self–self mentalization; i.e., metacognition), and can make inferences about how much other agents have insight into their own thought processes (i.e., other–self mentalization). These mentalizing processes are particularly important in navigating a variety of social environments and building successfully relationships. Here, we provide a brief overview of these three inferential processes in social interaction and provide some new definitions in order to clarify our approach.

Meta-cognition refers to our second order thoughts, that is, perceptions and beliefs about our own cognitive processes (Flavell, 1979; Nelson and Narens, 1990). This includes knowledge of our own beliefs, awareness of mental-states, and estimates of confidence in our abilities across different domains (Veenman et al., 2006; Rouault et al., 2018). To complement meta-cognition about one’s own cognitive processes, inferring the cognitive states of other individuals comes to bear in social contexts, and this has been referred to as mentalization (Frith and Frith, 2005).

It is of note that mentalization originally referred to cognitions about the mental states of both oneself and others (Premack and Woodruff, 1978). Thus, meta-cognition is historically a subcomponent of mentalization. Regardless of this historical relationship, it has been a recurring idea that meta-cognition is inherently a necessary aspect of inferring the mental states of others. For example, individuals with a higher capacity for self-reflection have been shown to have a higher capacity to understand others (Dimaggio et al., 2008). While this evidence suggests a common or overlapping mechanism, in this article, we refer to and argue for the utility of separating mentalization into two directional sub-components: self–other mentalization, and self–self mentalization.

A third, related component of social interaction is how much insight we think other agents have into our thoughts and intentions, hereby referred to as meta-mentalization, or other–self mentalization. In some respects, this can be viewed as a combination of perspective taking (self–other mentalization) and meta-cognition (self–self mentalization). The importance of meta-mentalization for strategic social interaction is relatively clear, for example in strategic decision making (Bhatt et al., 2010), a successful interaction requires real-time updating of the beliefs of others, and inference of how much the other player knows about their own thoughts (Silston et al., 2018). Notably, this meta-mentalization component can be influenced by two fundamental sources: estimates of another agent’s mentalizing ability, and estimates of your own ability to hide own thoughts to others (e.g., via faked external expressions). In the context of most social interactions, the influence of these sources ought to be negatively correlated – the better you think you are at deception, the less likely you think it is that someone else has true insight into your mental states, and vice-versa. Given the literature linking mentalization and meta-cognition, and theoretical accounts such as simulation theory (Gordon, 1986), it is highly likely that meta-mentalizing relies on the other two processes. That is, it in order to interrogate how another agent perceives you, it is first necessary to have a model of their beliefs, as well as your own.

There would be significant utility in defining a structure for, and outlining the relationships between meta-cognition, perspective taking, and meta-mentalization, both for clinical and healthy populations. The first step toward this would be the development of robust measures of these components. Indeed, efforts to develop such measures have previously been made, under various different theoretic views and validated on various samples. Most of these measures are interview-based, and have been developed with clinical applications in mind. These include the Reflective Function Scale (RFS; Fonagy et al., 1998) and the Parent Development Interview (Slade et al., 2004). Along similar lines, the Reflective Functioning Questionnaire (RFQ) developed by Fonagy et al. (2016) purports to measure mentalizing/meta-cognition capacity in both clinical and non-clinical samples, and was created for application in psychoanalysis and attachment theory. The Mentalization Scale (MentS), is another recently developed self-report measure (Dimitrijevic et al., 2018). While the latter scale purports to capture both mentalizing and meta-cognitive aspects, it does not address meta-mentalization. We feel that a comprehensive account of mentalizing, with respect to general interpersonal and social interactions, should necessarily include meta-mentalization, and ensure that (while it may be related to mentalizing and meta-cognition) meta-mentalization it is a distinct, measurable construct (Wu et al., 2020).

With the increase in the number of decision making studies involving social interaction, such as economic games (Frith and Singer, 2008; Polezzi et al., 2008), there is a greater requirement to measure aspects of mentalizing between interacting minds in everyday scenarios. For example, meta-mentalization is necessary for high level social interactions involving deception or trust, in which people not only need to have knowledge of themselves and knowledge of others, but also predictions of what others think about them (McCabe et al., 2003; Bhatt et al., 2010).

**FIGURE 1:**
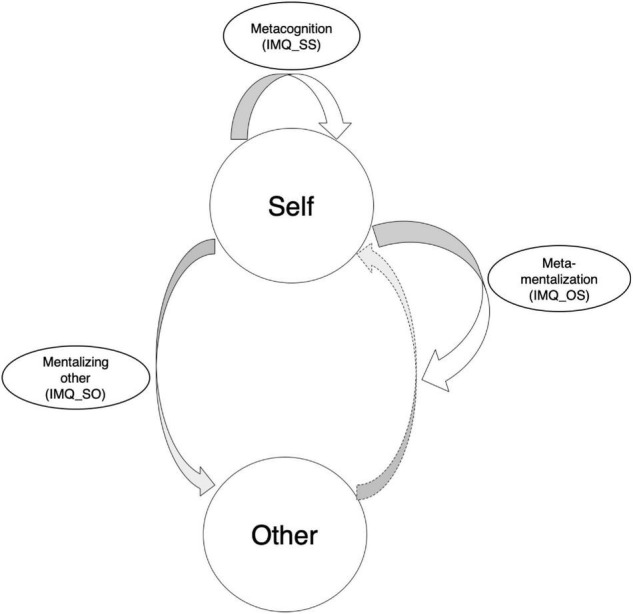
The three different components (IMQ_SS: self–self mentalization; IMQ_SO: self–other mentalization, and IMQ_OS: other–self mentalization) in our Interactive Mentalization Questionnaire.

In our theoretical framework, we aim to capture these aspects of mentalization in social interaction, and thus focus on these three components (self–other mentalization, self–self mentalization, and meta-mentalization, or other–self mentalization) (see Figure 1). We believe these constructs are fundamentally related, but independently measurable. Given that increasingly more studies place importance on decision making and social interaction, our goal was to develop an interactive mentalization questionnaire(IMQ) that would be specifically useful for capturing the following interactive mentalization components with three sub-scales:

(1) Mentalizing others: mentalization of other’s mental states from the perspective of the self (IMQ_SO; self–other);
(2) Meta-cognition: assessment of self-generated mental states from the perspective of the self (IMQ_SS; self–self);
(3) Meta-mentalization: evaluate mentalization of self-generated mental states from the perspective of others (IMQ_OS; other–self).

We hypothesized that these subscales would have predictive power with respect to players’ decisions in real online social interaction. Specifically, in light of simulation theory, we hypothesized that IMQ_SS (our measure of meta-cognition) would correlate with IMQ_SO (our measure of perspective taking), as well as IMQ_OS (our measure of meta-mentalizing). Given the previous study show mentalizing impairments in autisms, we also predicted negative correlations between the components in IMQ and autism spectrum quotient scores. Given that meta-cognition has been associated with decision confidence (Bang and Fleming, 2018), we further hypothesized that both the IMQ_SS and IMQ_OS would be positively associated with confidence ratings as measured in our version of the ultimatum game. Following this hypothesis, we also predicted that relative to those with lower meta-mentalization scores (IMQ_OS), individuals with higher scores who suffer social rejection will subsequently show lower happiness rating, given their higher expectations and self-confidence in their abilities.

## Study 1: Scale Development

### Method and Results

#### Participants and Procedure

All Mturk participants were recruited and provided informed consent according to the guidelines of the Institutional Review Board (Protocol number: 18-0790). 332 participants (38% female) recruited through Amazon Mechanical Turk (MTurk) (see Table 1). The instruction was “Please use the following scale to indicate your agreement with each of the questions.” “1 = very true for me 2 = somewhat true for me 3 = somewhat false for me 4 = very false for me”.

**TABLE 1:** The demographic characteristic of the samples.

Characteristic	Sample 1	Sample 2	Sample 3	Sample 4
*n*	332	417	450	299
Age range (years)	18∼65	18∼65	18∼65	18∼65
Mean age (years)	35.36	31.56	32.64	33.17
Female (%)	37.95	31.65	37.78	42.81

*n, Number of participants.*

#### Item Generation

We generated a pool of 24 items that were intended to reflect the mentalization of other’s mental states, one’s own mental states, and the assessment of how transparent these mental states are to others. The full list of 24 original items are shown in the Supplementary Table S1. All items were in a Likert-type format, with responses made on a 4-point response scale with 1 indicating strong agreement and 4 indicating strong disagreement. All items, together, were coded into a web-page formatted online survey (osf link: https://osf.io/2uarp/). Prior to analysis, we removed two items (6 and 11), due to a high degree of conceptual overlap with another item and a typographical error, respectively. The removal of these items did not substantially affect any of the analyses reported below.

**FIGURE 2:**
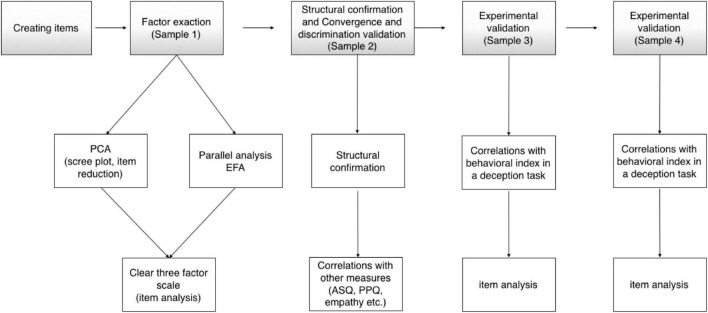
The flow chart depicting the processes to develop and validate IMQ.

A flow chart depicting the processes used to examine the validity of the IMQ is presented in Figure 2.

#### Exploratory Factor Analysis

We used the minimum residual (MinRes) method (Harman and Jones, 1966) for Exploratory Factor Analysis (EFA). The scree plots suggested the possibility of either three-factor or four-factor model (see Supplementary Figure S2). Given our aim to create a three-factor questionnaire, an EFA was performed specifying a three-factor solution. The results confirmed the factor structure. It revealed a root mean square of residuals (RMSR) of 0.04, under the standard 0.05 thresholds (Byrne, 1998; Diamantopoulos and Siguaw, 2000). The Tucker Lewis Index of factoring reliability was.89, RMSEA index was 0.06, and the Sample size adjusted Bayesian Information Criterion (BIC) was --607.08^1^.

#### Item Reduction

The Kaiser-Meyer-Olkin (KMO) test (Kaiser, 1974) statistic showed Measure of Sampling Adequacy (MSA) was 0.88, indicating suitability for PCA. The PCA analysis identified three factors that cumulatively explained 51.08% of the variance of responses (component 1: 31.14%, eigenvalue = 6.54; component 2: 13.84%, eigenvalue = 2.91; component 3: 6.11%, eigenvalue = 1.28) (see Supplementary Figure S1).

After reviewing the performance of each item in components, IMQ_3 showed poorly performed with low factor loading (overlapping factor loading: 0.38, 0.40, 0.31).

After deleting IMQ_3, we ran a second PCA and showed lower factor loading of one item (IMQ_19). We thus deleted the item and ran a final PCA, which did not identify low factor loading or double loading (difference lower than 0.1 between two factors). The following analyses were therefore based on the remaining 20 items (see Table 2).

**TABLE 2:** The IMQ scales: items, alpha reliabilities, standard deviations, and factor loadings.

Subscales and items	Factor loadings	Mean	*SD*	Skew	α if deleted
**IMQ_SO**					
*I believe that I am good at telling what another person is thinking.*	0.59	2.32	0.87	0.12	0.58
*I’m confident that I can tell what others are thinking.*	0.54	2.45	0.88	0.21	0.61
*When I watch a movie, I can always guess what the character will do next.*	0.38	2.38	0.91	0.19	0.59
*Under the right conditions, I’m good at lying to make people feel better.*	0.79	2.48	0.95	0.19	0.62
*I can tell someone one opinion, while thinking the opposite.*	0.70	2.4	0.94	0.17	0.6
*Compared to my friends (On average), I am better at guessing what others think.*	0.53	2.39	0.9	0.16	0.6
**IMQ_SS (metacognition)**					
*I have accurate insight into why I act the way I do.*	0.69	2.07	0.87	0.59	0.52
*I have accurate insight into why I think the way I do.*	0.64	2.13	0.85	0.53	0.53
*I can tell if others are teasing me.*	0.59	2.08	0.83	0.36	0.52
*When I fail, I know exactly why I failed.*	0.56	2.04	0.8	0.54	0.51
*Compared to my friends (On average), I have better insight into my own thoughts and behaviors.*	0.64	2.12	0.89	0.6	0.53
*I’m good at keeping my thoughts to myself.*	0.58	2.02	0.77	0.32	0.51
*I’m confident I’m correct when I perform a new task.*	0.49	2.18	0.82	0.35	0.55
*I have high confidence in knowing who I am.*	0.71	1.96	0.82	0.61	0.49
**IMQ_OS (meta-mentalization)**					
*Do you believe that some STRANGERS can read YOUR mind better than others?*	0.75	1.93	1.03	0.58	0.48
*Sometimes, I think people have direct insight into what I am thinking.*	0.69	2.31	1.04	0.15	0.58
*How confident are you that others can guess what you are thinking?*	0.71	2.38	1	0.14	0.59
*Do you believe in telepathy?*	0.71	2.21	1.09	0.29	0.55
*Advertisers are pretty accurate at knowing my current desires.*	0.81	2.45	0.99	0.03	0.61
*I cannot lie, because people will know my intentions.*	0.69	2.54	0.95	–0.11	0.63

*IMQ, Interactive Mentalization Questionnaire.*

#### Inter-Factor Correlations

A Pearson correlation analysis demonstrated significant relationships between the subscales of IMQ. Specifically, IMQ_SO was positively correlated with both IMQ_SS [*r*(332) = 0.45, *p* < 0.001] and negatively correlated with IMQ_OS [*r*(332) = –0.61, *p* < 0.001], with IMQ_OS was significantly negatively correlated IMQ_SS [*r*(332) = –0.25, *p* < 0.001].

#### Inter-Item Correlations

The average inter-item Pearson correlation was 0.49 for IMQ_OS, 0.32 for IMQ_SS, and 0.43 for IMQ_SO.

### Summary

Study 1 evaluated the factor structure and the psychometric properties of the IMQ. Overall, the PCA and EFA demonstrated a factor structure consistent with our proposal, the subscales showed adequate internal consistencies, and the relationships between the subscales and items did not show any statistical pathologies. The inter-factor and inter-item correlations indicated that the subscales appear to appropriately map onto separable components within a more general construct. Consistent with our proposed theoretical structure, the other–self mentalization (IMQ_OS) was correlated with the self–other mentalization (IMQ_SO), and weakly correlated with self–self mentalization (IMQ_SS).

## Study 2: Confirmatory Factor Analysis, Convergent and Discriminant Validity

The aim of Study 2 was to further confirm the factor structure of IMQ established in Study 1.

### Method

#### Participants and Procedure

The MTurk sample for Study 2 consisted of 417 participants (67.8% male; *M*age = 35.36, range 18–65 years old) (see Table 1). They were paid $1.5 for their participation.

#### Measures for Convergence and Discrimination Analysis

As a final item set had been established, the scales were administered along with other measures in order to establish convergent and discriminant validity. Measures used for this purpose included the following scales.

##### Autism Spectrum Quotient

The Autism Spectrum Quotient (ASQ) is a self-report scale designed to measure these traits (Baron-Cohen et al., 2001), and has been well validated, cross-culturally (Baron-Cohen et al., 2001, 2006). We expected moderately strong convergence between the ASQ and our subscales oriented to self-awareness (IMQ_SS), and a negative correlation with the subscale oriented to other’s mental states (IMQ_SO).

##### The Levenson Self-Report Psychopathy Scale Survey

The Levenson Self-Report Psychopathy Scale Survey (LSRP) is a scale (Levenson et al., 1995; Sellbom, 2011) to assess primary and secondary psychopathy (Miller et al., 2008; Wang et al., 2018), where primary psychopathy refers to selfish, uncaring, manipulative behavior toward others; and secondary psychopathy referring to impulsivity and other self-defeating behaviors. As previous work indicated metacognitive impairments and psychopathy in schizophrenia (Bo et al., 2014), we expected a strong association between LSRP and our subscales oriented toward the self (IMQ_SS and IMQ_OS).

##### Empathic Concern From Interpersonal Reactivity Index

The Interpersonal Reactivity Index (IRI) is a widely used scale to measure individual differences in empathy, and captures four separate aspects, including: (1) Perspective Taking (the tendency to spontaneously adopt the psychological point of view of others); (2) Fantasy (tendency to transpose themselves imaginatively into the feelings and actions of fictitious characters in books, movies, and plays); (3) Empathic Concern (EC: assesses “other-oriented” feelings of sympathy and concern for unfortunate others), and (4) Personal Distress (“self-oriented” feelings of personal anxiety and unease in tense interpersonal settings) (Davis, 1983). We used the EC to validate the IMQ subscales and expected a strong correlation between EC and our subscale for self–other mentalization (IMQ_SO).

##### Zimbardo Time Perspective Inventory

The Zimbardo Time Perspective Inventory (ZTPI) measures individual differences in time-orientation, with five subscales (Zimbardo and Boyd, 1999): (1) Past-Negative (a focus on events that went wrong in the past; (2) Present-Hedonistic (living in the moment – seeking pleasure, novelty, and sensation, and avoiding pain); (3) Present-Fatalistic (feeling that decisions are moot because predetermined fate plays the guiding role in life, e.g., “what will be, will be”), (4) Past-Positive (a focus on the “good old days,” e.g., keeping scrapbooks, collecting photos, and looking forward to celebrating traditional holidays), and (5) Future (simply planning for the future and trusting that decisions will work out). We used the Future subscale to validate the IMQ_OS and IMQ_SS, as it measures people’s confidence about their decisions or plans for future, which ought to be related to the meta-cognition and meta-mentalization components (Stolarski and Witowska, 2017).

#### Confirmatory Factor Analysis

Dimensionality of the IMQ was evaluated using the PCA and factoring method described in Study 1. Before proceeding with the factor analysis, the KMO factor adequacy test showed MSA = 0.86.

With the ‘lavaan’ CFA function in the R (Rosseel, 2012), we used the NLMINB optimization method, with a maximum likelihood (ML) estimator, and 39 iterations for confirmatory factor analysis (CFA). The fit of the model was assessed through the following indices: (1) the Satorra Bentler scaled chi-square (χ2); (2) the comparative fit index (CFI); (3) the goodness-of-fit index (GFI); and (4) the root mean square error of approximation (RMSEA) (Browne and Cudeck, 1992).

### Results

#### Confirmation of Factor Structure

The CFA indicated satisfactory results with respect to a three-factor model [GFI = 0.915, CFI = 0.914; Tucker-Lewis Index (TLI) = 0.902, RMSEA = 0.057, and the χ^2^(167) = 393.044, *p* < 0.001].

#### Correlations With the Other Measures in Sample 2

Correlations between the IMQ subscales and the other measures are presented in Table 3 (n = 417).

**TABLE 3:** Correlations of IMQ subscales with other measures: Sample 2.

Scale	IMQ_OS	IMQ_SS	IMQ_SO
IMQ_SS	–0.16		
IMQ_SO	−0.48****	0.55****	
LSRP	−0.57****	−0.18*	0.24****
LSRP_primary	−0.53****	–0.10	0.27****
LSRP_secondary	−0.53****	−0.28****	0.15
**ZTPI**			
Past_negative	−0.32****	–0.15	0.16
Future	0.13	0.31****	0.08
Past_positive	–0.13	0.24***	0.13
Present_hedonism	−0.51****	0.06	0.32****
Present_fatalism	−0.58****	−0.18*	0.21**
IRI*_*EC	0.15	0.15	–0.05
ASQ	−0.31****	−0.42****	−0.19**

*LSRP, the Levenson Self-Report Psychopathy Scale Survey; ZTPI, Zimbardo Time Perspective Inventory; IRI_EC, Empathic concern from Interpersonal Reactivity Index; ASQ, Autism Spectrum Quotient.*

*Corrected p < 0.05; **Corrected p < 0.01; ***Corrected p < 0.001; ****Corrected p < 0.0001.

The ASQ score was strongly negatively correlated with three IMQ subscales, *r* = –0.31, *p* < 0.001 for IMQ_OS, *r* = –0.42, *p* < 0.001 for IMQ_SS, *r* = –0.19, *p* < 0.01 for IMQ_SO. This pattern supports the notion that those with better capacity in all three mentalization domains are less likely to exhibit autism traits.

The IMQ_OS was negatively correlated with psychopathy scores in the LSRP, *r* = –0.57, *p* < 0.001, and IMQ_SS were negatively correlated with psychopathy scores in the LSRP, *r* = –0.18, *p* < 0.05. In contrast, we observed a positive correlation between IMQ_SO and LSRP psychopathy, *r* = 0.24, *p* < 0.001.

With regard to the time perspective scale, the Past Negative Hedonism (*r* = –0.32, *p* < 0.001), Present Hedonism (*r* = –0.51, *p* < 0.001) and Present Fatalism subscales (*r* = –0.58, *p* < 0.001) were negatively correlated with IMQ_OS. The Present Hedonism (*r* = 0.32, *p* < 0.001) and Present Fatalism (*r* = 0.21, *p* < 0.001) were all positively correlated with IMQ_SO. Moreover, the Future subscale (*r* = 0.31, *p* < 0.001) and Past Positive (*r* = 0.24, *p* < 0.001) strongly positively correlated with IMQ_SS.

However, the EC – as measured by the IRI – did not show significant correlation with IMQ subscales after correction.

In line with our hypotheses, these relationships imply that our subscales capture aspects of meta-cognition (e.g., a positive correlation with future confidence), can reflect social competence (a negative correlation with ASQ), and yet are divergent from others measures such as EC.

#### Inter-Factor Correlations

IMQ_SO was positively correlated with IMQ_SS (*r* = 0.55, *p* < 0.001), and IMQ_SO was negatively correlated with IMQ_OS (*r* = –0.48, *p* < 0.001), while IMQ_OS was negatively correlated with IMQ_SS (*r* = –0.16, *p* < 0.001).

#### Cronbach’s α

The internal consistencies of the three subscales were 0.81 for IMQ_OS, 0.83 for IMQ_SS, and 0.76 for IMQ_SO.

#### Inter-Item Correlations

The average inter-item Pearson correlation was 0.42 for IMQ_OS, 0.37 for IMQ_SS, and 0.35 for IMQ_SO.

Overall, the data from the CFA further validated the three-factor model of the IMQ. Moreover, the convergent and discriminant validity indicated that the IMQ_OS and IMQ_SO scales are related to, but also distinct from alternative measures, such as EC and ASQ.

In sum, Study 2 further supported our three-factor measurement scale by replicating the results of Study 1, while in addition providing a comparison with related measures.

## Study 3: Interactive Mentalization Questionnaire Subscales and the Deception Task

To further validate the IMQ scale, we collected data from a task involving mentalizing and spontaneous deception which often occur in strategic social interactions. In our ultimatum game paradigm (Kirk et al., 2011; Marchetti et al., 2011), one player (the proposer) is given a sum of money and then must choose how much to tell and offer to the other player (the responder). The responder may accept or reject the offered amount, with rejection leading to both players receiving nothing. In this task, individuals require mentalization in order to form expectations and predictions. Therefore, a successful strategy relies on a player’s confidence about their own beliefs, the content of their opponent’s beliefs, and their opponent’s specific beliefs about the player’s own thoughts.

### Method

#### Participants and Procedure

The sample consisted of 450 Mturk participants (*M*age = 32.64, range from 18 to 65 years old, 62.2% male). They were paid $0.5 for their participation and paid with the payoff in the game after finished the whole task.

Given the nature of the task (see below), participants were assigned to play the role of a “proposer” or a “responder.” Thus, the sample was ultimately divided into 218 “proposers” (*M*age = 33.28, *SD* = 9.61, 61.46% male) and 232 “responders” (*M*age = 32.05, *SD* = 9.2, 62.06% male).

#### Experimental Task

Our task was based on a UG task with asymmetric information (Vesely, 2014), previously used to examine self-interest driven dishonesty. In this version, only the proposer knows the initial endowment, and has an opportunity to tell the responder how much this amount is. They can either be honest to report the true amount, or dishonest, and report any other amount. Our version of the task was a one-shot game (i.e., there was only one round and participants did not switch roles, leaving no possibility of strategic behavior based on/due to learning).

**FIGURE 3:**
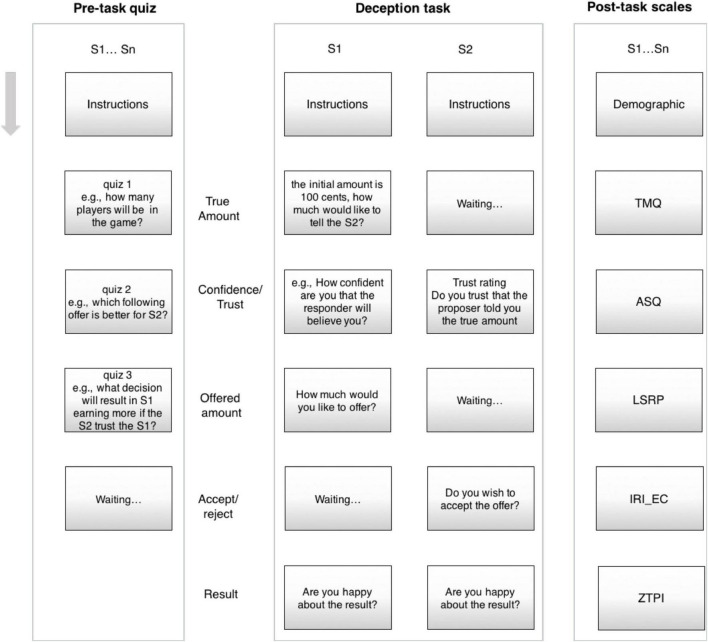
The plot of the MTurk paradigm run in sample 3.

Two participants were randomly paired over internet and assigned a role of either proposer or responder. The participants were first shown detailed instructions about the task (Figure 3). On the next page, the endowment – randomly chosen from a range of 30–160 cents – was shown to the proposer. This amount was not shown to the responder. On the same page, the proposer was prompted to tell the responder how much this initial endowment was (Notably, the larger the initial endowment was, the more opportunity for deception in this phase of the task.). On the subsequent page, the proposer was asked to rate how confident they were that the proposer would believe the amount stated as the initial endowment. Simultaneously, the responder was asked to rate how much they trusted that the stated amount corresponded with the true endowment. Both of these ratings were on a five-point scale. Next, the proposer was prompted to actually offer a proportion of the initial endowment. On the penultimate page, the responder was prompted to either accept or reject this offer. Finally, the true initial endowment, the offered amount, and the payoff (based on the responder’s decision) were displayed on the screen, and both players were asked to rate how happy they were with the outcome on a five-point scale. The experiment was followed by questionnaires measuring mentalization (IMQ), psychopathy (LSRP), empathy (IRI_EC), time perspective (ZTPI), and autism traits (ASQ).

### Data Analysis

To test the stability of the IMQ structure when delivered in the context of real social interaction, we repeated CFA implemented with the same approach as in Study 2.

In order to examine the relationship between the subscales of the IMQ and the different behavioral measures of mentalization taken during the task, we constructed a number of indices for each role. For the proposer data, we first defined the *deception index* as the total amount minus the told amount, divided by the total amount (i.e., the fraction of the initial endowment potentially “kept for oneself”). Secondly, we defined *self-aware fairness* as the offered amount as a fraction of the total amount. Thirdly, for both proposer and responder, we defined *other-aware fairness* as offered amount as a fraction of the told amount. Given our interest in meta-mentalization, we were also interested in the *proposer’s confidence* about their decision, and the *responder’s trust* rating.

Given the hypothesized relevance of all of these indices to the subscales of the IMQ, we generated a Pearson correlation matrix for these measures. Furthermore, we also included alternative questionnaires (ASQ, IRI_EC, LSRP, and ZTPI) in order to replicate the results from Study 2, and to identify whether these measures were also related to our behavioral indices.

Lastly, hierarchical multiple regression was performed to further investigate whether and to what degree the subscales of the IMQ were able to capture variance in the decisions and behavioral indices of the proposers and responders.

### Results

#### Confirmatory Factor Analysis

Confirmatory factor analysis indicated a good fit for a three-factor model [GFI = 0.886, CFI was 0.89; TLI = 0.87, RMSEA = 0.073, and the χ2 (167) = 563.91, *p* < 0.001].

#### Inter-Item Correlations

The average inter-item-correlation was 0.47 for IMQ_OS, 0.42 for IMQ_SS, and 0.40 for IMQ_SO.

#### Behavioral Indices

A one-sample *t*-test demonstrated that the deception *index* was significantly greater than zero [*t*(217) = 5.4, *p* < 0.001, Cohen’s *d* = 0.37, 95% CI = 0.10–0.22], implying that proposers were dishonest on average, and confirming that the context of the task was able to drive dishonest behavior. Notably, we found substantial individual differences in dishonesty – the mean *deception index* was 0.16, with a standard deviation of 0.4.

Another description of dishonesty was the *other-aware fairness* (*M* = 0.66, *SD* = 0.27) was significantly higher than *self-aware fairness* (*M* = 0.55, *SD* = 0.4) [*t*(217) = 4.52, *p* < 0.001, Cohen’s *d* = 0.60, 95% CI = 0.05–0.17], again implying that proposers were dishonest in general.

Proposers showed relatively high confidence (*M* = 3.70, *SD* = 0.89, where 1 was “very unconfident” and 5 was “very confident”), and relatively high happiness about the result (*M* = 3.53, *SD* = 1.26. where 1 was “very unhappy” and 5 was “very happy”). For the proposers whose offers were accepted, happiness ratings were significantly higher (*M* = 4.23, *SD* = 0.82) relative to those whose offers were rejected (*M* = 2.03, *SD* = 1.49), [*t*(33) = 8.01, *p* < 0.001, Cohen’s *d* = 2.32, 95% CI = 1.64–2.76].

A majority of responders (84%) accepted the offered amount, with moderate ratings of trust (*M* = 3.14, *SD* = 1.21). For the responders who accepted the offer, happiness ratings were significantly higher (*M* = 3.77, *SD* = 1.11) relative to when offers were rejected (*M* = 2.29, *SD* = 1.29), [*t*(47) = 6.50, *p* < 0.001, Cohen’s *d* = 1.01, 95% CI = 1.01–1.93].

#### Interactive Mentalization Questionnaire Scores for Different Roles/Players Conditional by Accept/Reject Response

To examine whether IMQ scores were significantly different for proposers and responders, conditional on whether the offers were rejected or accepted, we performed several *t*-tests. Firstly, for proposers, there were no significant differences in total IMQ scores [*t*(37) = 0.85, *p* = 0.40] as a function of outcome, nor were there any differences between the three subscales [IMQ_OS: *t*(38) = 0.17, *p* = 0.86; IMQ_OS: *t*(40) = 0.17, *p* = 0.86; IMQ_SS: *t*(39) = 1.12, *p* = 0.27]. For responders, total IMQ scores did not differ significantly on the basis of rejection (*M* = 56.05, *SD* = 7.02) or acceptance [*M* = 55.46, *SD* = 6.45; *t*(48) = 0.85, *p* = 0.63]. Neither the IMQ_SO nor IMQ_SS subscales showed any significant difference between the rejected offer responders (IMQ_SO: *M* = 15.88, *SD* = 3.83; IMQ_SS: *M* = 23.43, *SD* = 4.64) vs. the accepted offer responders [IMQ_SO: *M* = 14.78, *SD* = 3.96, *t*(50) = 1.54, *p* = 0.13; IMQ_SS: *M* = 23.35, *SD* = 4.95, *t*(49) = 0.08, *p* = 0.93]. However, IMQ_OS scores were significantly higher for responders who rejected the offer (*M* = 17.92, *SD* = 4.20) than those who accepted the offer [*M* = 16.15, *SD* = 3.34; *t*(47) = 6.50, *p* = 0.007, Cohen’s *d* = 0.43, 95% CI = –1.69 to 1.84], suggesting that higher meta-mentalization capacity was associated with an increase in rejections.

#### Relationship Between Interactive Mentalization Questionnaire Subscales and Behavioral Indices

The first exploratory analysis of proposers’ data indicated that proposer’s meta-mentalization was negatively associated with the confidence in the deception task, and the fairness level of the allocation (Table 4). However, the *deception index* was not associated with IMQ scores (Table 4). For the responders’ data, we observed a significant negative correlation between IMQ_OS and the trust rating to the proposer (*r* = –0.28, *p* < 0.01, corrected). We also found a negative correlation between IMQ_OS score and happiness ratings (*r* = –0.31, *p* < 0.01, corrected).

**TABLE 4:** Correlations of IMQ subscales with other measures: Sample 3.

Index or Scale	IMQ_OS	IMQ_SS	IMQ_SO
***Proposers’ data***			
Proposers.IMQ_SS	–0.06		
Proposers.IMQ_SO	−0.44****	0.40****	
Proposers.deception index	0.09	0.08	0.00
Proposers.otheraware_fairness	−0.38****	–0.15	0.13
Proposers.selfaware_fairness	−0.27*	–0.10	0.08
Confidence	−0.25*	0.03	0.01
Results_happy	–0.06	–0.03	–0.05
LSRP	−0.53****	–0.21	0.20
Proposers. LSRP_primary	−0.51****	–0.19	0.19
Proposers. LSRP_secondary	−0.50****	–0.20	0.19
Proposers.past_negative	−0.41****	–0.08	0.23
Proposers.future	0.27*	0.34****	0.06
Proposers.past_positive	–0.05	0.27*	0.04
Proposers.present_hedonism	−0.45****	0.03	0.32****
Proposers.present_fatalism	−0.64****	–0.18	0.28**
EC	0.27*	0.21	–0.02
AQ	−0.27*	−0.55****	–0.24
***Responders’ data***			
Responders.IMQ_SS	−0.26*		
Responders.IMQ_SO	−0.66****	0.55****	
Responder_trust	−0.28**	–0.09	0.15
Proposers.otheraware_fairness	–0.07	0.02	0.07
Proposers.selfaware_fairness	–0.08	0.05	0.06
Results_happy	−0.31***	0.02	0.18
LSRP	−0.56****	–0.20	0.29**
Responders.LSRP_primary	−0.55****	–0.12	0.31***
Responders.LSRP_secondary	−0.48****	−0.29**	0.19
Responders.past_negative	−0.36****	–0.10	0.31***
Responders.future	0.02	0.37****	0.12
Responders.past_positive	–0.18	0.27*	0.27*
Responders.present_hedonism	−0.49****	0.16	0.44****
Present_fatalism	−0.54****	–0.13	0.37****
IRI_EC	0.14	0.07	–0.07
ASQ	–0.11	−0.47****	−0.29**

*LSRP, the Levenson Self-Report Psychopathy Scale Survey; ZTPI, Zimbardo Time Perspective Inventory; IRI_EC, Empathic concern from Interpersonal Reactivity Index; ASQ, Autism Spectrum Quotient.*

*Corrected p < 0.05; **Corrected p < 0.01; ***Corrected p < 0.001; ****Corrected p < 0.0001.

To investigate whether the questionnaire score, deception index, confidence and outcome were associated with the outcome happiness rating, we ran a GLM, using the *deception index*, confidence, IMQ_SO, IMQ_OS, IMQ_SS and offer response (accept vs. reject) as predictors for proposers’ happiness ratings. The results showed significant effects for confidence (β = 0.86, *SE* = 0.32, *p* = 0.007), and offer response (β = 2.26, *SE* = 0.18, *p* < 0.001), but not for the IMQ subscales. Given our specific hypothesis that higher confidence in meta-mentalizing might interact with the response to the offer, we ran a GLM using only IMQ_OS and offer response (accept vs. reject) as predictors, we found that an interaction between IMQ_OS and offer response was a significant predictor (β = 0.14, *SE* = 0.04, *p* < 0.001). We further analyzed this interaction by evaluating simple slopes (Aiken and West, 1991). When the offer was accepted, the slope of the regression line of IMQ_OS was not significant (β = 0.01, *SE* = 0.02, *t* = 0.62, *p* = 0.53), while the slope of the regression line of IMQ_OS was significant when the offer was rejected (β = –0.12, *SE* = 0.03, *t* = –3.54, *p* < 0.01) (see Supplementary Figure S3).

#### Correlations Between Interactive Mentalization Questionnaire and Other Measures

Both the proposers and responders showed similar correlations with other questionnaire measures as in Study 2. For example, we replicated the negative correlations between IMQ_OS and psychopathy (measured by LSRP) both for proposers (*r* = –0.53, *p* < 0.001) and responders (*r* = –0.56, *p* < 0.001). We also reproduced the negative correlation between IMQ components and autism traits (for proposers, IMQ_OS to ASQ: *r* = –0.27, *p* < 0.05; IMQ_SS to ASQ: *r* = –0.55, *p* < 0.001; for responders, IMQ_SS *to* ASQ: *r* = –0.47, *p* < 0.001; IMQ_SO to ASQ: *r* = –0.29, *p* < 0.01), with the highest correlation with IMQ_SS.

## Study 4: Confirmation the Validity of the Interactive Mentalization Questionnaire in the Deception Task

One potential criticism of Study 3 was that most offers were accepted prior to the IMQ measurement, which may affect the scores in the IMQ. We wanted to further to validate the IMQ when implemented exclusively after unsuccessful social interactions with others. Thus, in Study 4 we implemented same online task, but this time we manipulated the task such that each participant was assigned to the role of the proposer, and all offers were artificially rejected. Our specific aims here were twofold: (1) to validate the IMQ in a different social context; and (2) to examine any possible state-dependency of the IMQ subscales. With regard to the latter, we hypothesized that the subscales related to mentalizing and meta-mentalizing would be relatively state-dependent (i.e., sensitive to the social environment), while the subscale related to self-awareness would be relatively stable.

### Methods

#### Participants and Procedure

Two hundred and twenty nine participants (*M*age = 32.64, range from 18 to 65 years old, 62.2% male) were again recruited through MTurk, were paid $1.5 for their participation.

#### Experimental Task

The task in Study 4 was almost identical to that of Study 3, with the exception that all players were assigned to the role of the proposer, and all offers were ultimately rejected in order to replicate the IMQ results after unsuccessful social interaction.

We showed participants the same instructions as in Study 3, in order to make the players believe they were interacting with another player. As in Study 3, the proposer was given an endowment (from 30 to 160 cents), prompted to report the endowment to the other player, asked to rate their confidence (*1* = *not confident at all* to *100* = *super confident*) that their report was believable, prompted to make an offer to the responder, and finally rate their happiness (*1* = *not happy at all* to *100* = *super happy*) with the outcome. The task was again followed by questionnaires (IMQ, LSRP, IRI_EC, ZTPI, and ASQ).

#### Data Analysis

First, CFA was implemented with the same approach as in Study 2.

As in Study 3, we constructed behavioral indices (naturally only for the proposer role), and generated a Pearson correlation matrix between these indices, the subscales of the IMQ, and the alternative questionnaire measures.

Regression analysis was performed to investigate whether and to what degree the subscales of the IMQ were able to capture variance in the decisions and behavioral indices of the proposers.

To explore the possible effect of previous social interaction context on the IMQ subscales, we compared IMQ scores from Study 4 with the scores from Study 3, conditioned on accepted offers. The distribution plots of the IMQ sub scores are shown in Supplementary Figure S5.

### Results

#### Confirmatory Factor Analysis

Confirmatory factor analysis indicated a good fit for a three-factor model [GFI = 0.879, CFI = 0.872; TLI = 0.854, RMSEA = 0.07, and the χ2(167) = 426.27, *p* < 0.001].

#### Behavioral Indices

Consistent with the result of Study 3, proposers were in general dishonest in reporting the endowment [*t*(298) = 13.96, *p* < 0.001, Cohen’s *d* = 0.81, 95% CI = 0.18–0.24].

Furthermore, other-aware fairness (*M* = 0.64, *SD* = 0.35) was significantly higher than self-aware fairness (*M* = 0.48, *SD* = 0.24), [*t*(298) = 10.01, *p* < 0.001, Cohen’s *d* = 0.58, 95% CI = 0.13–0.19]. Out of the 1–100 rating slide, the proposers showed relative high confidence (very unconfident 1–100 very confident), *M* = 69.14, *SD* = 24.60, and unhappy about the being rejected result, *M* = 30.63, *SD* = 36.54.

#### Interactive Mentalization Questionnaire Scores After Successful and Unsuccessful Interaction

There were no significant differences in the scores for either the IMQ_SO nor IMQ_SS subscales between Study3 and Study 4 (both *p* > 0.12). However, we did find a significant difference of the meta-mentalization component between the two studies for the IMQ_OS [*t*(399) = –2.86, *p* = 0.004, Cohen’s *d* = –0.25, 95% CI = – 1.92 to –0.36). That is, the proposers who had their offer accepted in Study 3 (*M* = 16.19, *SD* = 4.25) exhibited lower IMQ_OS than the proposers who were rejected in Study 4 (*M* = 17.33, *SD* = 4.31), suggesting some influence of context on this measure.

#### Relationship Between Interactive Mentalization Questionnaire Subscales and Behavioral Indices

We first wished to confirm our hypothesis that IMQ_OS should be correlated with the proposer’s confidence in the interaction. As in Study 3, the results indicated that proposers’ meta-mentalization was negatively associated with confidence ratings (*r* = –0.21, corrected *p* < 0.01), other-aware fairness ratings (*r* = –0.37, corrected *p* < 0.01), and self-aware fairness ratings (*r* = –0.34, corrected *p* < 0.01) (Table 5).

**TABLE 5:** Correlations between questionnaires and behavioral index in Sample 4.

Scale	IMQ_OS	IMQ_SS	IMQ_SO
***Proposers’ data***			
IMQ_SS	0.02		
IMQ_SO	−0.34****	0.50****	
Total IMQ	0.51****	0.76****	0.51****
Deception index	–0.08	0.01	0.13
Otheraware_fairness	−0.37****	–0.06	0.15
Selfaware_fairness	−0.34****	–0.07	0.07
Confidence	−0.21*	0.03	0.12
Results_happy	−0.51****	–0.06	0.19
**LSRP**			
LSRP_primary	−0.49****	–0.07	0.31****
LSRP_secondary	−0.59****	−0.24**	0.19***
**ZTPI**			
Past_negative	−0.39****	–0.12	0.21*
Future	0.22*	0.36****	0.06
Past_positive	–0.07	0.27****	0.17**
Present_hedonism	−0.48****	0.02	0.38****
Present_fatalism	−0.63****	–0.10	0.26***
IRI_EC	0.06	0.13	0.02
ASQ	−0.34****	−0.45****	−0.26**

*LSRP, the Levenson Self-Report Psychopathy Scale Survey; ZTPI, Zimbardo Time Perspective Inventory; IRI_EC, Empathic concern from Interpersonal Reactivity Index; ASQ, Autism Spectrum Quotient.*

*Corrected p < 0.05; **Corrected p < 0.01; ***Corrected p < 0.001; ****Corrected p < 0.0001.

In a GLM using the *deception index*, confidence, IMQ_SO, IMQ_OS, and IMQ_SS as predictors for proposers’ happiness ratings, the results revealed a significant effect of the confidence ratings (β = 0.98, *SE* = 0.42, *p* < 0.001), but not for the IMQ subscales (IMQ_SO: β = –0.04, *SE* = 0.02, *p* = 0.21, IMQ_OS: β = 0.17, *SE* = 0.09, *p* = 0.06, IMQ_SS: β = 0.003, *SE* = 0.02, *p* = 0.87). In the GLM, we also found a significant interaction effect between confidence and IMQ_OS (β = –0.05, *SE* = 0.02, *p* < 0.001). It indicated when the confidence was high, the slope of the regression line of IMQ_OS was significant (β = –0.06, *SE* = 0.03, *t* = –2.13, *p* = 0.03), while the slope of the regression line of IMQ_OS was not significant when the confidence was medium (β = –0.02, *SE* = 0.02, *t* = –0.81, *p* = 0.42) or low (β = –0.03, *SE* = 0.03, *t* = 0.86, *p* = 0.39) (see Supplementary Figure S4).

#### Correlations Between Interactive Mentalization Questionnaire and Other Measures

Both the proposers and responders showed similar correlations with other questionnaire measures as in Study 2 and Study 3 (Table 5). For example, we replicated the negative correlations between IMQ_OS, IMQ_SS and psychopathy measured by LSRP (IMQ_OS and LSRP primary: *r* = –0.49 *p* < 0.001, IMQ_OS and LSRP secondary: *r* = –0.59, *p* < 0.001, IMQ_SS and LSRP secondary: *r* = –0.24, *p* < 0.01, IMQ_SO and LSRP primary: *r* = 0.31, *p* < 0.001, IMQ_SO and LSRP secondary: *r* = 0.19, *p* < 0.001). Again, IMQ components were negatively correlated with autism traits, with highest correlation with IMQ_SS (IMQ_OS and ASQ: *r* = –0.34, *p* < 0.001; IMQ_SS and ASQ: *r* = –0.45, *p* < 0.001; IMQ_SO and ASQ: *r* = –0.26, *p* < 0.01).

## Discussion

### General Discussion and Conclusions

Our aim was to develop and validate a new, brief self-report measure to assess individual differences in three psychological components of interactive mentalizing. These include measures that reflect: (i) the capacity to infer the mental states and thoughts of others (IMQ_SO), (ii) the ability to look inward to monitor and assess one’s own thought processes (e.g., IMQ_SS), and (iii) beliefs about the transparency of one’s own thoughts to others (IMQ_OS). To achieve this aim, this work was structured in four major parts. First, we developed sets of questionnaire items that reflected the three kinds of psychological components that should be theoretically linked, and explored the structure scale of the scale (Figure 1). We next used an independent sample to confirm the questionnaire structure and correlated the subscale scores with theoretically related alternative measures. We then used the subscales to assess behavioral decisions in a social interaction game, as well as to assess individual confidence ratings – ecologically valid social measures. Finally, we used the subscales to replicate these results in the context of unsuccessful mentalizing context. Taken together, these studies provide initial support for the structure of the proposed IMQ scale, and indicate a reliable measurement of individual differences in mentalization processes oriented to oneself and to others.

### Structure and Hierarchy in Interactive Mentalization Questionnaire

In our model of IMQ, the three components are related to, but also independent from each other. With respect to the correlations among three components, the results indicate that the measure of IMQ_SS (self–self mentalization/meta-cognition) was negatively correlated with the IMQ_OS (other–self mentalization/meta-mentalization), but positively correlated with IMQ_SO (self–other mentalization).

The positive correlation between the IMQ_SS and the IMQ_SO subscales is consistent with simulation theory (Harris, 1992; Carruthers, 1996), which hypothesizes that people rely on meta-cognitive processes to model the mental states of others. Support for this theory comes from the proposed role of “mirror neurons,” which are involved both in self-generated processes, as well as during the observation of the same actions generated by others (Gallese and Goldman, 1998). The positive correlation between IMQ_SS and IMQ_SO fits the notion that people rely on similar meta-level thinking for the inference of both our own and other’s abilities, beliefs, and emotions.

Across our different samples, we consistently observed that the IMQ_OS and IMQ_SO subscales were negatively correlated. While our original hypotheses proposed a relationship between these two subscales and reported confidence independently, we did not anticipate a direct relationship between them. One possible explanation, however, is that some individuals overestimate their own abilities relative to others (Taylor and Brown, 1988; Kruger and Dunning, 1999). This would lead to them expressing better self-assessment of perspective taking (IMQ_SO) as well as better self-assessment of their ability to hide their thoughts from others (note that IMQ_OS is reverse scored, such that higher scores indicate less transparency of one’s own thoughts to others). Interestingly, while both of these subscales ought to contribute to higher social competence, such an overestimation of one’s own abilities has been shown to be detrimental for social interaction (i.e., “tooting one’s own horn”; Colvin et al., 1995). It is difficult to provide much support for this interpretation without non-self-report assessments of social competence with which to contrast to our self-report measure. One more speculative possibility is that there is a shared and limited resource for meta-cognition, such that those who think more about others think less about themselves and vice-versa. However, we know of no current evidence that would support this viewpoint.

While an ideal measure of these components of social cognition ought to be consistent, we also acknowledge that individuals dynamically learn and adjust their beliefs about themselves and others over time. This should be particularly apparent in ongoing social interaction, or under different social contexts. While we did not observe state-based changes in our measures, *per se*, we did observe a gross change in the IMQ_OS subscale under different social contexts. In Study 3, IMQ_OS scores were higher after a cohesive social interaction (offer acceptance) than in Study 4, after social rejection. Given that the IMQ_OS subscale theoretically reflects how well other individuals can infer one’s own beliefs and motivations, it makes sense that this should be affected after an unsuccessful social interaction. An interesting further question is whether behavioral changes, such as differences in expression, might occur as a result of discrepancies in other-self mentalization. Further studies measuring body language (facial expressions, gestures, speech patterns) may be able to address this.

### Correlations With Other Measures

With regard to the relatively rich literature on the relationships between mentalization and other traits, we wanted to ensure our scale captured some of these existing relationships, while accounting for enough new variation to be valuable on its own. Across three studies, we observed that IMQ_SO was positively correlated with psychopathy, while IMQ_OS and IMQ_SS were both negatively correlated with psychopathy. These results are not entirely consistent with some previous studies that failed to find a relationship between psychopathy and theory of mind (Richell et al., 2003; Del Gaizo and Falkenbach, 2008), or literature demonstrating a negative association between psychopathy and mentalization (Choi-Kain and Gunderson, 2008; Bateman et al., 2013). However, it is important to note that our study focused on typical individuals with and trait-psychopathy, rather than clinically determined psychopaths. Notably, our results partially consistent with other findings showing different components of psychopathy show different relationships with mentalization, such that antisocial psychopaths are associated with lower mentalization ability, while interpersonal psychopaths are associated with higher mentalization ability (Sandvik et al., 2014). In another study, within a non-clinical sample, psychopathy was shown to be negatively correlated with overall accuracy in an emotion expression test (Ali and Chamorro-Premuzic, 2010; Vonk et al., 2015).

Consistent with literature pointing to impairments of meta-cognition and mentalization in individuals with autistic traits (Baron-Cohen et al., 1986; Zalla et al., 2015), and our hypotheses, we observed negative correlations between IMQ components and autistic traits across our studies. More specifically, our results showed that autistic traits are most strongly correlated with the IMQ_SS component, and most weakly correlated with the IMQ_SO. The latter result in particular is consistent with findings indicating that individuals high in autism traits are unable to recognize their own emotions and find it difficult to identify their own thoughts (Baron-Cohen, 1997). One interesting avenue for future research is to identify whether our IMQ subscales can provide a more tailored fingerprint of autistic traits and symptoms, particularly during development. For example, it may be possible that the different IMQ components may map onto different symptoms, and different degrees of dysfunctional behavior and social functioning, and this could provide a valuable method for psychiatric classification.

We hypothesized that as it relates to decision confidence about future plans, the future component of the ZTPI would be associated with IMQ_SS and IMQ_OS – measures that require an estimate of confidence or ability. Across our studies, our results showed that future perspective was strongly positively correlated with IMQ_SS and weakly positively correlated with IMQ_OS, but that there was no association with IMQ_SO. This generally supports our hypothesis that decision confidence should be related to self-assessments of ability. In relation to the overconfidence interpretation of the negative correlation between IMQ_SO and IMQ_OS above, there is some literature that reports that increases in construal (psychological distance) increase self-idealization (Kivetz and Tyler, 2007). Interestingly, these increases in construal, which can be predictions about future actions or outcomes, enhance not only self-idealization and overconfidence, but also overconfidence in the abilities of others (Griffin et al., 1990; Vallone et al., 1990). This contrasts with the negative correlation between IMQ_SO and IMQ_OS, which seems to show that given this level of construal, individuals may still preference their own abilities above those of others. To our knowledge this has not been directly shown, but is supported by our findings.

### Correlations With Behaviors

With respect to the proposers in Study 3, our results showed that the IMQ_OS subscale was negatively correlated with self-reported fairness and confidence ratings when proposers were informing the responder about the total amount on offer. While the relationship with confidence ratings makes sense with respect to how the individuals feel about their own strategic deception abilities, the relationship with self-reported fairness appears to imply that these individuals felt some superiority over their opponents. That is, individuals were more likely to believe that the portioned rewards were fairer if they thought that their opponents had poor insight into their own decisions.

As for the responder data, their trust of the proposer was negatively correlated with their IMQ_OS score, and positively correlated with their IMQ_SO score. These latter results seem to indicate a tradeoff where individuals who rated the proposer’s capacity for insight as inferior were also less likely to trust them, while if they rated their own mentalization capacity as higher, they were more likely to trust them. A simple explanation for the first relationship is that expectations of ability are generalized, so that if individuals think that another agent has poor ability to have insight into their own mental states, they also have poor social abilities in general – including trustworthiness. One possible interpretation for the second relationship is provided by a social projection account, whereby individuals use beliefs about how they would react in the same situation in order to identify with, and place trust in the decisions of others. Thus, people’s expectations about the trustworthiness of others are correlated with estimates of their own tendency to trust others (Thielmann and Hilbig, 2014). One good example of this is in the Trust Game: one player (the investor) decides how much money out of an initial endowment to send to another player (the trustee). The sent amount is then tripled, and the trustee decides how much of the money received to send back to the investor). A study using this paradigm found that selfish investors with good mindreading skills were less likely to display trust, and invested less, than those with worse mindreading skills (Derks et al., 2015). Overall, these results demonstrate that our measures are an appropriate tool to capture aspects of behavior in real social interactions.

Notably, we did not find the direct correlation between deception index and proposers’ IMQ subscales scores, but we did find deception-related results with IMQ subscale scores. As in our task, we found dishonesty for most participants, and we tried to not only capture the deception index for the proposers, but also to ask the proposer and responder to rate their feelings of confidence and happiness about the results. First, since most of the participants lied in the task, we observed a significant negative correlation between IMQ_OS and the trust rating to the proposer. Further, our results showed IMQ_OS scores were significantly higher for responders who rejected the offer than those who accepted the offer, suggesting that higher meta-mentalization capacity was associated with an increase in rejections. It may mean that people with higher meta-mentalization score can recognize deception more and reject the more. As for the proposer’s data, we found proposer’s meta-mentalization was negatively associated with the confidence in the deception task, and the fairness level of the allocation. This correlation between IMQ OS subscales scores and confidence in the deception task, may indicate that people lied (lower fairness level) but with lower confidence in the deception. In summary, our results provide evidence between IMQ subscale scores and deception from other aspects (trust, rejection decisions, deception confidence) but not the deception index directly.

### Broader Issues and Future Directions

There are several limitations to our studies. One concern is the ecological validity of our online deception task, i.e., difference between online testing and lab testing. Deception can be induced by different motivations, in both MTurk and lab settings (Greene and Paxton, 2009; Wu et al., 2009; Suri et al., 2011; Fischbacher and Föllmi-Heusi, 2013; Cui et al., 2018). Participants in Study 3 and Study 4 acted deceitfully toward other online players with monetary incentivization. We note that many morally relevant decisions may be different when they are interacting with real people in the lab (Levitt and List, 2007). While our results from this task fit our theoretical hypotheses, it remains to be tested if these extend to other, face-to-face interactive scenarios. It is also difficult to provide support for a clear interpretation of the negative correlation between IMQ_OS and IMQ_SO, while future studies, perhaps using computational modeling, may give more insights on this topic.

A further line of enquiry focuses on the implications of mentalization for different kinds of populations. One example is an investigation of typical or atypical development in meta-cognition and its impact on different aspects of social functions throughout the lifespan. Similarly, it would be of great interest to test social decision making and mentalization processes in subclinical and clinical samples (Sharp and Venta, 2012; Specht et al., 2016).

Despite limitations to this early piece of work, the IMQ developed and validated here provides a valuable empirical tool for addressing these issues in future research.

## Data Availability Statement

The original contributions presented in the study are included in the article/Supplementary Material, further inquiries can be directed to the corresponding author/s.

## Ethics Statement

The studies involving human participants were reviewed and approved by Caltech (Protocol Number: 18-0790). The patients/participants provided their written informed consent to participate in this study.

## Author Contributions

HW and DM: designed the research. HW: performed the research and analyzed the data. HW, BF, and DM: wrote the manuscript.

## Conflict of Interest

The authors declare that the research was conducted in the absence of any commercial or financial relationships that could be construed as a potential conflict of interest.

## Publisher’s Note

All claims expressed in this article are solely those of the authors and do not necessarily represent those of their affiliated organizations, or those of the publisher, the editors and the reviewers. Any product that may be evaluated in this article, or claim that may be made by its manufacturer, is not guaranteed or endorsed by the publisher.

## Footnotes

1 The alternative four-factor model EFA results showed root mean square of residuals (RMSR) was 0.04. The Tucker Lewis Index of factoring reliability is 0.92, RMSEA index is 0.053, BIC = –586.69. The fit results seem good for four-factor model as well, with however, only two items for one of the components.

## Supplementary Material

Supplementary Table S1 The full items of the original items.

Supplementary Figure S1 The screen plot of the PCA components in sample 1.

Supplementary Figure S2 The parallel analysis scree plots in sample 1.

Supplementary Figure S3 The prediction effect of interaction between offer response and meta-mentalization in sample 3.

Supplementary Figure S4 The prediction effect of interaction between confidence and meta-mentalization on happiness of the results.

Supplementary Figure S5 The distributions of three sub scales in sample 1, sample 2, sample 3, and sample 4, the Shapiro–Wilk normality test cannot reject the non-normal hypothesis for all scores.
