# Supplementary material for: Mentalizing During Social Interaction: The Development and Validation of the Interactive Mentalizing Questionnaire
Source: Front Psychol. 2022 Feb 17;12:791835. doi: 10.3389/fpsyg.2021.791835 (PMC8891136; doi:10.3389/fpsyg.2021.791835)
Supplement: Supplementary Table S1 — The full items of the original items. [file Data_Sheet_1.docx]

# Supplemental materials

Table S1 the full items of the original items

| *1.     Do you believe that STRANGERS can read YOUR mind better than others?* |
| --- |
| *2.     I have accurate insight into why I act the way I do* |
| *3.     My thoughts are private and no one, but me, has access to them.* |
| *4.     I believe that I am good at telling what another person is thinking* |
| *5.     I’m confident that I can tell what others are thinking.* |
| *6.     I am rarely confident in my decisions* |
| *7.     When I watch a movie, I can always guess what the character will do next* |
| *8.     Sometimes, I think people have direct insight into what I am thinking* |
| *9.     Under the right conditions, I’m good at lying make people feel better.* |
| *10.  I can tell someone one opinion, while thinking the opposite.* |
| *11.  My thoughts often come out of know where* |
| *12.  How confident are you that others can guess what you are thinking?* |
| *13.  Compared to my friends (On average), I am better at guessing what others think.* |
| *14.  I have accurate insight into why I think the way I do* |
| *15.  I cannot lie, because people will know my intentions.* |
| *16.  Advertisers are pretty accurate at knowing my current desires.* |
| *17.  I can tell if others are teasing me.* |
| *18.  When I fail, I know exactly why I failed* |
| *19.  If I find someone attractive, I believe that they know I find them attractive.* |
| *20.  Compared to my friends (On average), I have better insight into my own thoughts and behaviors.* |
| *21.  I’m good at keeping my thoughts to myself.* |
| *22.  Do you believe in telepathy?* |
| *23.  I’m confident I’m correct when I perform a new task* |
| *24.  I have high confidence in knowing who I am.* |

IMQ_2, IMQ_3, IMQ_4, IMQ_5, IMQ_7, IMQ_9, IMQ_10, IMQ_13,IMQ_14,

IMQ_17, IMQ_18 ,IMQ_20, IMQ_21, IMQ_23, IMQ_24 are reversal scoring.

Figures

Figure S1.


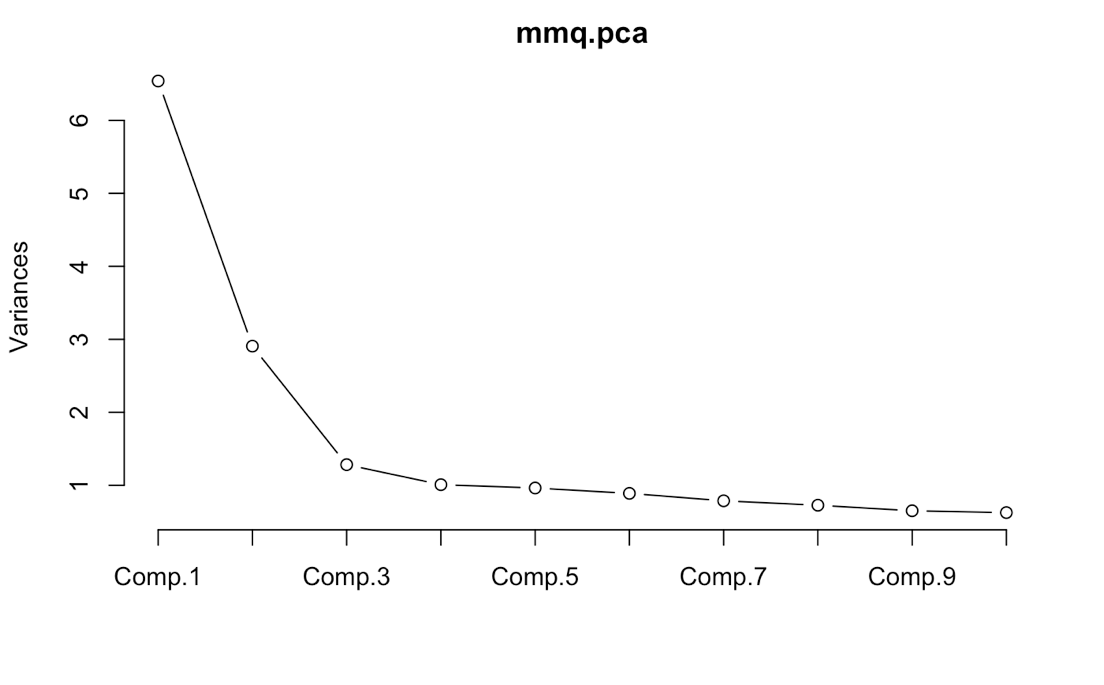


*Figure S1.* The screen plot of the PCA components in sample 1

Figure S2.


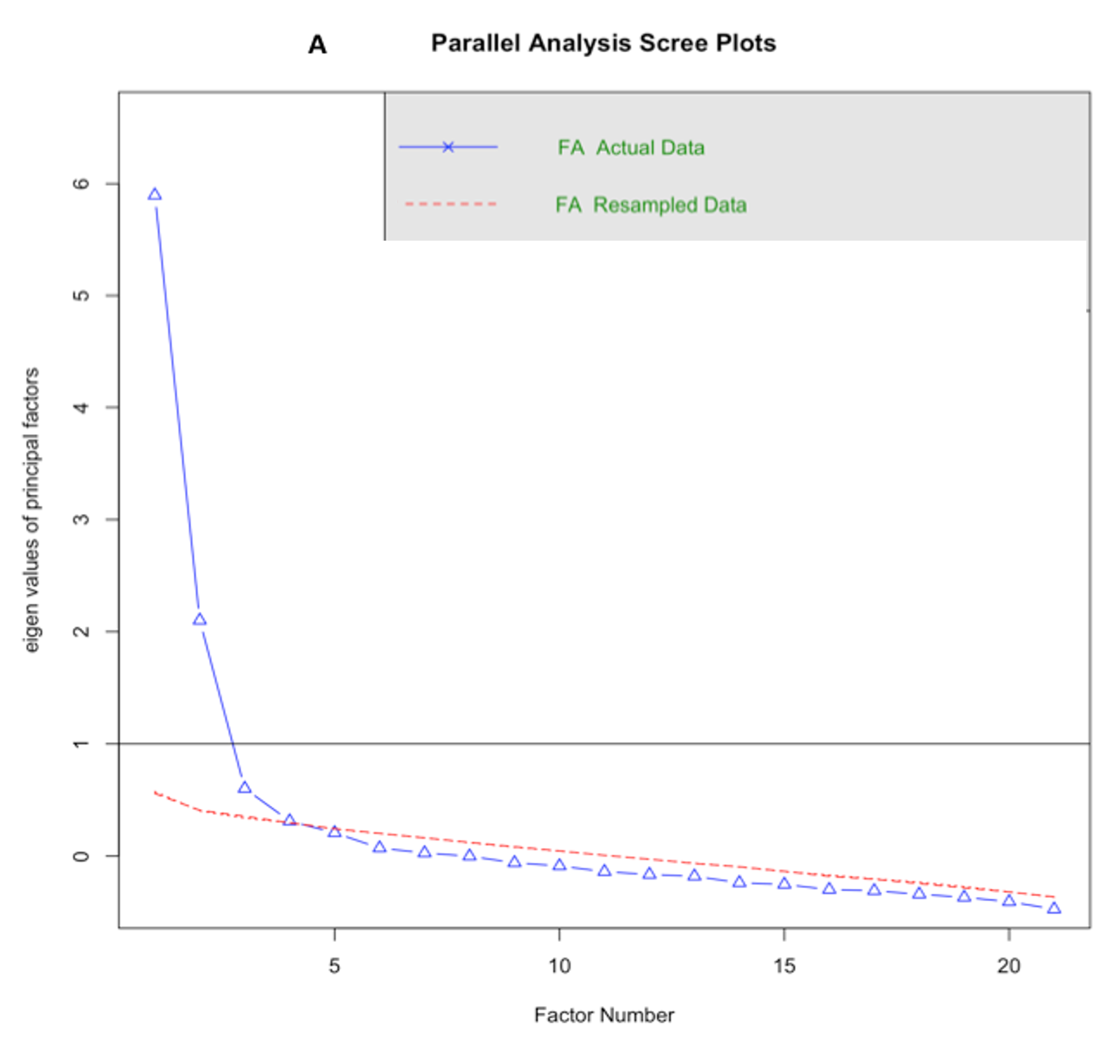


*Figure S2.* The parallel analysis scree plots in sample 1

Figure S3.


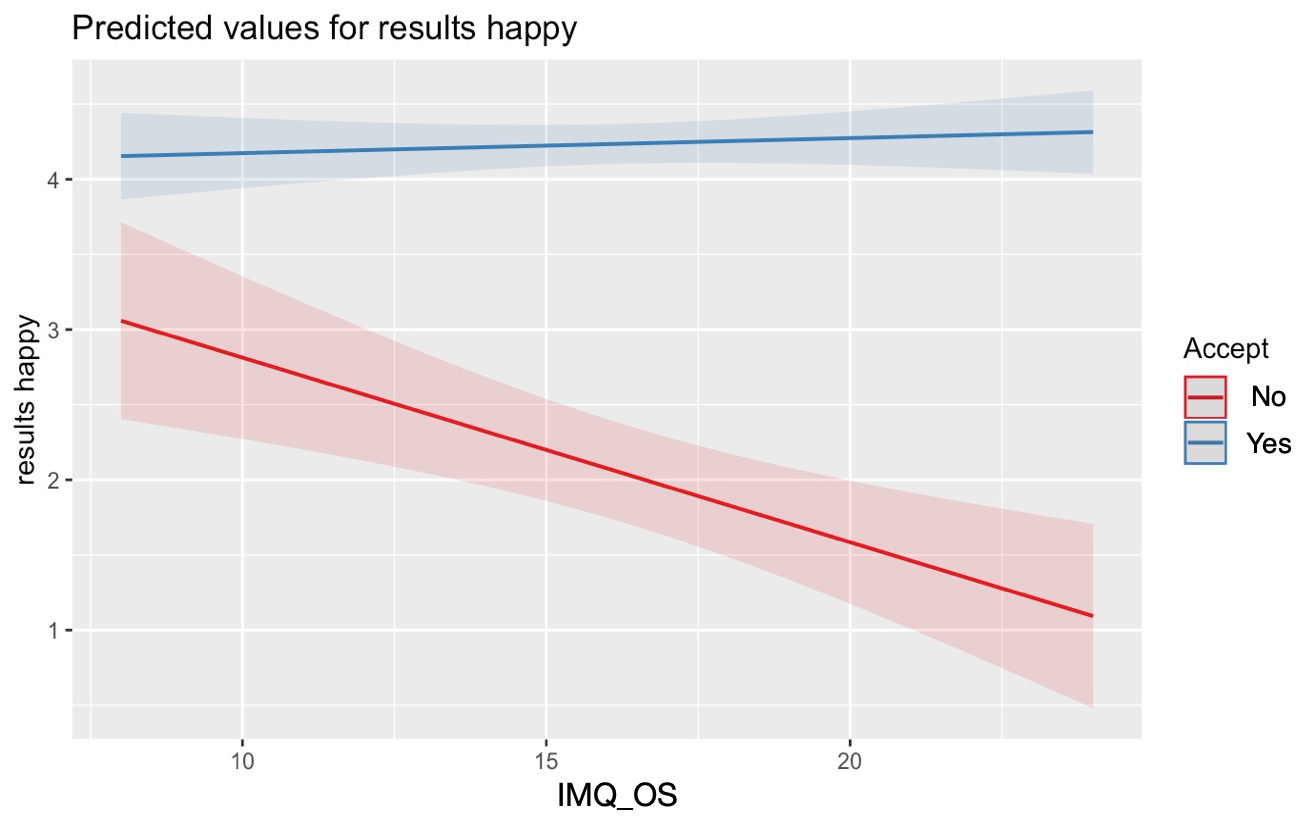


Figure S3 the prediction effect of interaction between offer response and meta-mentalization in sample 3

Figure S4.


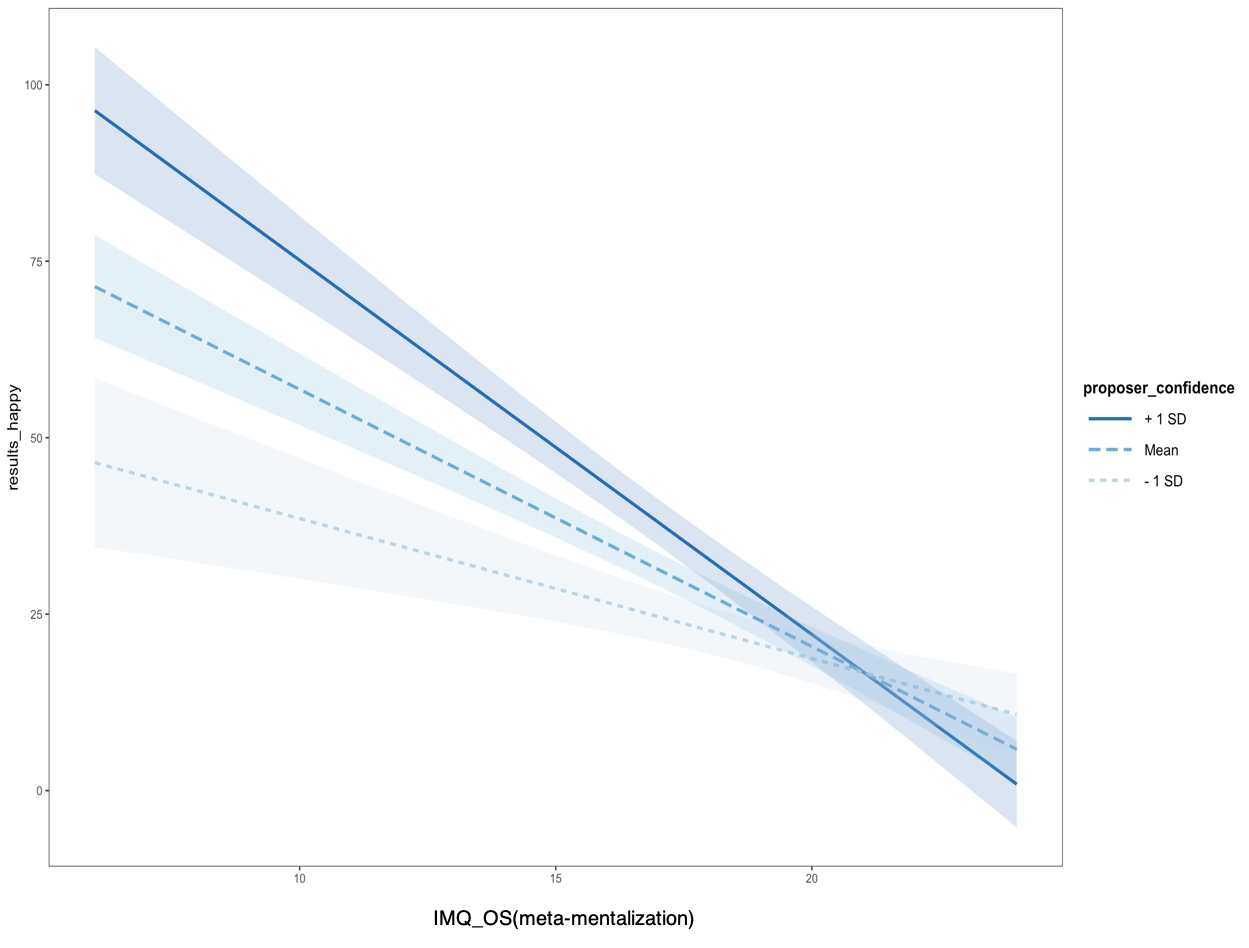


*Figure S4.* the prediction effect of interaction between confidence and meta-mentalization on happiness of the results

Figure S5.


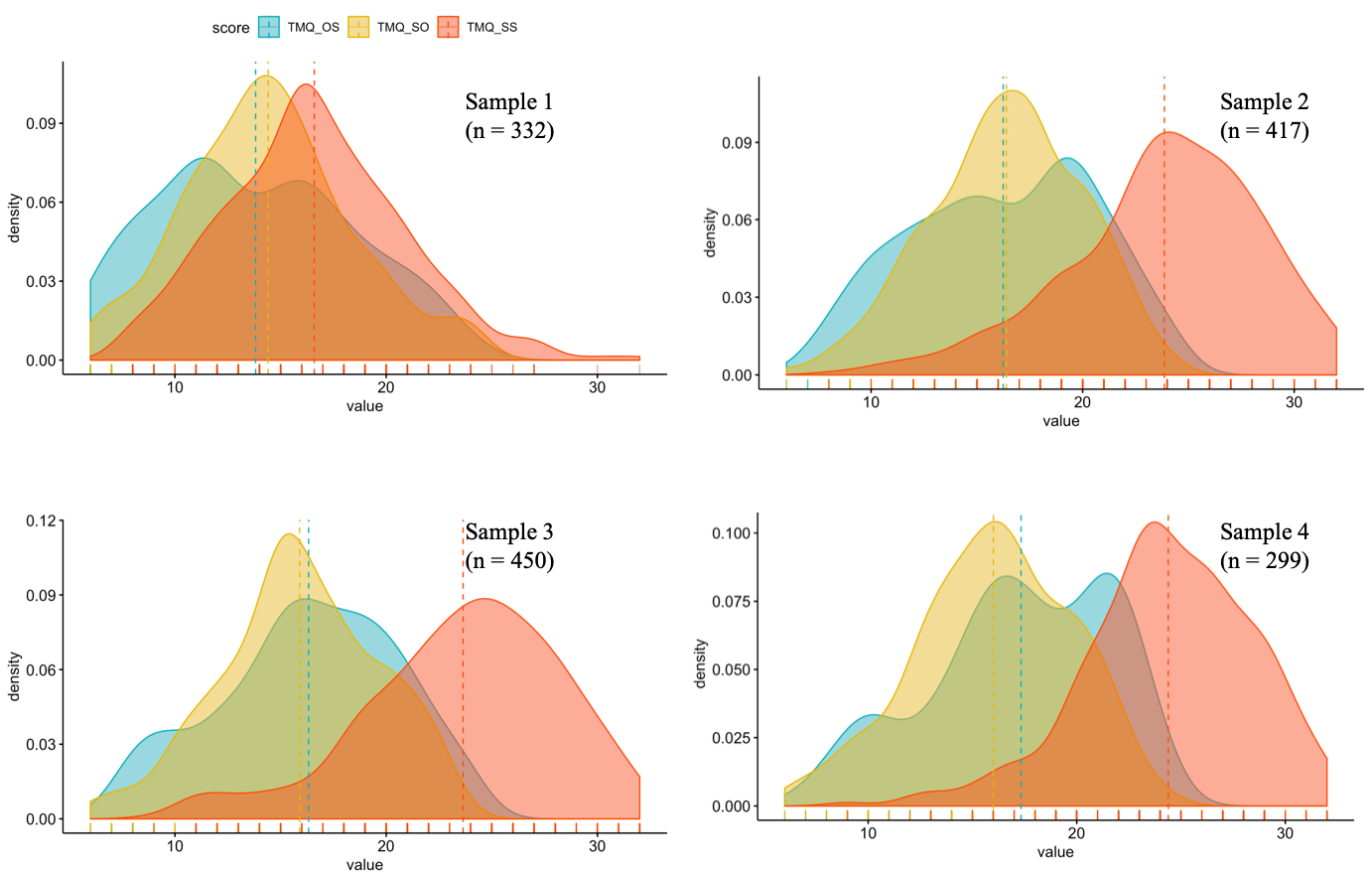


*Figure S5.* the distributions of three sub scales in sample 1, sample 2, sample 3 and sample 4, the Shapiro-Wilk normality test cannot reject the non-normal hypothesis for all scores.
